# EPSPS Gene Copy Number and Whole-Plant Glyphosate Resistance Level in *Kochia scoparia*

**DOI:** 10.1371/journal.pone.0168295

**Published:** 2016-12-16

**Authors:** Todd A. Gaines, Abigail L. Barker, Eric L. Patterson, Philip Westra, Eric P. Westra, Robert G. Wilson, Prashant Jha, Vipan Kumar, Andrew R. Kniss

**Affiliations:** 1 Department of Bioagricultural Sciences and Pest Management, Colorado State University, Fort Collins, Colorado, United States of America; 2 Department of Agronomy and Horticulture, University of Nebraska, Scottsbluff, Nebraska, United States of America; 3 Southern Agricultural Research Center, Montana State University, Huntley, Montana, United States of America; 4 Department of Plant Sciences, University of Wyoming, Laramie, Wyoming, United States of America; Agriculture and Agri-Food Canada, CANADA

## Abstract

Glyphosate-resistant (GR) *Kochia scoparia* has evolved in dryland chemical fallow systems throughout North America and the mechanism of resistance involves 5-enolpyruvylshikimate-3-phosphate synthase (*EPSPS*) gene duplication. Agricultural fields in four states were surveyed for *K*. *scoparia* in 2013 and tested for glyphosate-resistance level and *EPSPS* gene copy number. Glyphosate resistance was confirmed in *K*. *scoparia* populations collected from sugarbeet fields in Colorado, Wyoming, and Nebraska, and Montana. Glyphosate resistance was also confirmed in *K*. *scoparia* accessions collected from wheat-fallow fields in Montana. All GR samples had increased *EPSPS* gene copy number, with median population values up to 11 from sugarbeet fields and up to 13 in Montana wheat-fallow fields. The results indicate that glyphosate susceptibility can be accurately diagnosed using *EPSPS* gene copy number.

## Introduction

*Kochia scoparia* (L.) Schrad. is a competitive weed that can cause substantial yield loss, and is particularly a problem weed in sugarbeet and chemical fallow [1, 2]. *K*. *scoparia* is a C4 summer annual broadleaf weed that can germinate and emerge early in the growing season and is tolerant to heat, drought, and saline conditions [1]. *K*. *scoparia* has protogynous flowers in which the stigmas usually emerge one week before pollen is shed and are receptive to foreign pollen which can promote outcrossing between plants in close proximity [3]. It also produces copious amounts of pollen for extended periods of time, which is generally an indication that the species is naturally highly outcrossing [1]. *K*. *scoparia* stem breakage at the soil surface during senescence allows for a tumbling seed dispersal mechanism that can contribute to high rates of spread in the western US [4]. In Wyoming, *K*. *scoparia* densities as low as 0.2 plants m^-1^ of crop row reduced sugarbeet root yield by 18% [5]. The outcrossing nature of *K*. *scoparia* combined with prolific seed production results in genetically diverse populations that facilitate the evolution of herbicide resistance [6].

GR *K*. *scoparia* was first identified in Kansas [7] and has now been identified in multiple Great Plains States including Colorado, South Dakota, North Dakota [8], Montana [9], Nebraska [10], and in the Canadian provinces of Alberta [11], Saskatchewan and Manitoba [12]. Most of the reported GR *K*. *scoparia* populations appear to have evolved in reduced- or no-till chemical fallow systems [13], where glyphosate is used as the primary weed control practice during fallow periods. Glyphosate resistance in *K*. *scoparia* has been attributed to gene duplication in which resistant plants contain 3 to 10 times more functional copies of the gene encoding 5-enolpyruvylshikimate-3-phosphate synthase (*EPSPS*) [8]. These extra gene copies result in overproduction of the EPSPS enzyme, which is the target enzyme inhibited by glyphosate. High-coverage sequencing analysis of *EPSPS* transcripts from glyphosate-resistant *K*. *scoparia* revealed the absence of any known resistance-conferring non-synonymous mutations [8], further demonstrating that increased EPSPS expression due to *EPSPS* gene duplication and increased transcription confers glyphosate-resistance in *K*. *scoparia*. Increased *EPSPS* gene copy number and expression has been shown to be a mechanism for glyphosate-resistance in *K*. *scoparia* collected from Kansas [14]; Colorado, North Dakota, and South Dakota [8]; and Montana [15]. Detection of *EPSPS* genes on distal ends of homologous chromosomes suggests that increase in *EPSPS* gene copies in GR *K*. *scoparia* occurred as a result of unequal crossover during meiosis resulting in tandem gene duplication [16]. The extra *EPSPS* copies are stably inherited in *K*. *scoparia*, consistent with the cytogenetic observation that the extra *EPSPS* copies are located at a single locus [16]. Analysis of *EPSPS* gene copy number and resistance level in *K*. *scoparia* populations from Kansas suggest that there has been a progressive increase in *EPSPS* gene copies and level of glyphosate resistance over time from 2007 to 2012 [16]. In two GR *K*. *scoparia* populations from Kansas, *EPSPS* gene copy number was correlated to resistance level, such that within a resistant *K*. *scoparia* population, individuals with higher *EPSPS* copy number displayed less injury symptoms compared to individuals with lower *EPSPS* copy number [14].

Besides wheat-chemical fallow rotation, widespread adoption of GR sugarbeet systems in the US has resulted in significant glyphosate selection pressure, and increasingly sugarbeet growers are reporting reduced *K*. *scoparia* control with glyphosate. Adoption of glyphosate-resistant (GR) sugarbeet systems has resulted in improved weed control and reduced sugarbeet injury compared to conventional sugarbeet systems [17]. Glyphosate can provide weed control similar to or greater than conventional weed control programs consisting of three applications of desmedipham, phenmedipham, triflusulfuron, and clopyralid [18]. Prior to commercial introduction, net economic return was predicted to be significantly greater for GR sugarbeet systems compared to conventional sugarbeet due to reduced crop injury and better weed control [17]. GR sugarbeets were commercially introduced in 2007. By 2009, more than 85% of US sugarbeet hectares were seeded with GR cultivars, with remaining areas seeded with conventional cultivars that had resistance to specific pests or diseases that were not commercially available with the GR trait [19]. Sugarbeet growers have significantly reduced tillage and increased net economic return since adoption of GR sugarbeet [20].

Therefore, the objectives of this study were to a) compare the level of glyphosate resistance in *K*. *scoparia* collected from sugarbeet fields and wheat-chemical fallow fields; b) determine whether GR *K*. *scoparia* from sugarbeet fields has the same mechanism of resistance (increased *EPSPS* gene copy number) as identified in dryland chemical fallow-based systems; and c) quantify the effect of *EPSPS* copy number on whole-plant response to glyphosate across numerous (65) *K*. *scoparia* accessions collected from sugarbeet fields from Colorado, Wyoming, Nebraska, and Montana and 12 accessions collected from wheat-chemical fallow fields from Montana.

## Materials and Methods

### Plant material

In the autumn of 2013, 65 sugarbeet fields from Colorado, Wyoming, Nebraska, and Montana were surveyed by Western Sugar Cooperative agricultural staff for surviving *K*. *scoparia* plants, referred to as the Western Sugar (WS) accessions. The samples for the study were collected from private land. The authors confirm that all owners of each field site gave permission to collect samples for the study from their site. Seed was collected from maturing *K*. *scoparia* plants within the sugarbeet fields, or in some cases, along field margins. GPS coordinates were recorded for each sampling site (S2 Data). Seed was stripped by hand from multiple branches of each *K*. *scoparia* plant and placed in a plastic bag. Each seed sample (accession) represented one to five individuals, and was therefore not necessarily representative of the entire *K*. *scoparia* population from a given field. This survey was biased for surviving *K*. *scoparia* plants from fields where glyphosate was used. Seed from each site was sent to the Panhandle Research & Extension Center in Scottsbluff, Nebraska for whole-plant dose-response bioassays. Once received, all accessions were air dried and cleaned before use in the dose-response studies.

A second collection of *K*. *scoparia* seed was conducted in Montana, referred to as the Montana (MT) accessions. Seeds were collected from survived *K*. *scoparia* plants from 12 different chemical fallow fields (wheat-chemical fallow rotation) in Hill, Liberty, and Toole Counties, Montana, USA in autumn 2013. At each site, *K*. *scoparia* seeds were collected from 5 to 10 randomly selected plants. Seeds from those plants in a field were combined into a composite sample (accession). The sampled fields had a history of repeated glyphosate use (at least three applications of glyphosate, each at 870 g ae ha^-1^) per year for weed control during the chemical fallow period (preceding winter wheat) over more than 6 years. The whole-plant dose-response studies for the MT *K*. *scoparia* accessions were conducted in the greenhouse at the Montana State University—Southern Agricultural Research Center near Huntley, Montana. For all seed collections, the growers/land owners provided the permission to enter their fields and collect *K*. *scoparia* seeds. We also acknowledge that the research did not involve any endangered or protected species.

### Greenhouse bioassay

Each *K*. *scoparia* accession was screened for susceptibility to glyphosate. For the initial set of *K*. *scoparia* accessions, approximately 15 to 20 seeds were planted in 10 cm ×10 cm plastic pots filled with a 50:50 by weight mixture of field soil and commercial potting mix. After planting, pots were placed in the greenhouses at the two sites (Montana and Nebraska), where air temperature was maintained at 27 C and pots were watered several times per day with an automated sprinkler system. *K*. *scoparia* emerged approximately three days after planting, and the pots were thinned to three plants per pot shortly after emergence. When *K*. *scoparia* averaged 10 cm in height, each accession was treated with different rates of glyphosate (Roundup PowerMAX, Monsanto Company) along with ammonium sulfate (2% w/v) and nonionic surfactant (0.25% v/v). A nontreated control was also included for each accession. For the WS accessions, glyphosate was applied at 0, 435, 870, 1740, and 3480, and 5050 g ae ha^-1^. For the MT accessions, glyphosate was applied at 0, 217, 435, 870, 1740, 3480, 5220, 6960, and 8700 g ae ha^-1^. Each glyphosate rate by accession interaction was replicated six times for a total of 36 pots per accession, with three individual plants in each pot. A total of 85 *K*. *scoparia* accessions were included in the greenhouse bioassays. Herbicide treatments were applied in a CO_2_-pressurized moving-nozzle spray chamber calibrated to deliver 224 L ha^-1^ spray solution.

For both sets of bioassay experiments, *K*. *scoparia* injury was evaluated visually 14 days after treatment on a scale from 0 to 100 where 0 represented no injury and 100 represented death of all plants in the pot. For statistical analysis, injury evaluations were converted to a binomial response of alive (<95% injury) or dead (≥95% injury). A two-parameter log-logistic model (Eq 1) appropriate for binomial data (alive vs dead) was used to estimate the glyphosate dose causing 50% mortality (LD_50_) for each *K*. *scoparia* accession. The log-logistic model is of the form:

$$Y=1/(1+\left({\text{e}}^{b}{}^{*(\text{log}\left(x\right)-\text{log}(LD50)}\right)$$ (1)

Where *Y* is the probability of survival; *x* is the glyphosate dose in g ae ha^-1^; and *b* is the slope of the curve at the LD_50_.

### *EPSPS* gene copy number assay

Based on the results of the greenhouse bioassay, a sub-set of 40 *K*. *scoparia* WS accessions and 12 MT accessions that exhibited a range of whole-plant resistance levels were assayed for *EPSPS* copy number at Colorado State University and Montana State University, respectively, using a common protocol. To determine *EPSPS* copy number in these *K*. *scoparia* accessions, genomic DNA was extracted from individual plants using the DNeasy Plant Mini Kit (Qiagen). Young leaf tissue (100 mg) was sampled from 6–12 plants for each accession when the plants reached 7–10 cm in height. Samples were disrupted in 2 mL tubes using the TissueLyser II (Qiagen). The extraction proceeded using the standard DNeasy Plant Mini Kit protocol. Genomic DNA was eluted in 50 μL of 37 C HPLC water. The concentration and quality of the gDNA were determined using a NanoDrop 1000.

*EPSPS* copy number was estimated using quantitative PCR (qPCR) on the genomic DNA with previously reported primers [8]. Acetolactate synthase (*ALS*) was used as a normalization gene because *ALS* copy number has been shown to not vary among *K*. *scoparia* individuals [8]. Each reaction contained 12.5 μL of PerfeCTa SYBR^®^ green Super Mix (Quanta Biosciences), 1 μL of the forward and reverse primers [10 μM final concentration], and 5 ng gDNA in a total volume of 25 μL. A BioRad CFX Connect Real-Time System was used for all qPCR. The temperature profile was as follows: 3 min at 95 C followed by 40 rounds of 95 C for 30 sec, 60 C for 30 sec, and 72 C for 30 sec, with a fluorescence reading taken after each round. A melt curve from 65–95 C in 0.5 C increments was performed with a fluorescence reading after each increment to determine the number of PCR products formed in each reaction. Only single PCR products were observed in melt-curve analysis from *EPSPS* and *ALS* primers as expected in all samples, indicating PCR amplification of only the intended genes occurred. The cycle was recorded for each sample at which the fluorescence reading crossed a threshold (C_t_) indicating exponential increase, and relative *EPSPS* gene copy number was calculated using the comparative C_t_ method [21] as 2^ΔCt^ (ΔC_t_ = C_t_^ALS^− C_t_^EPSPS^) [8]. For each *K*. *scoparia* accession, 6 to 12 samples were measured for relative *EPSPS* copy number, and the mean, standard error of the mean, and median *EPSPS* copy number were calculated for each accession.

### Effect of *EPSPS* copy number on whole-plant response

The relationship between LD_50_ from the greenhouse bioassay and the median *EPSPS* copy number was of interest. Regression techniques were used to develop an empirical model that predicts the whole plant response based on the number of *EPSPS* gene copies; methods and results from this analysis have been provided as supplementary information (S1 File; S1 and S2 Figs).

## Results and Discussion

### Greenhouse bioassay

**Fig 1:**
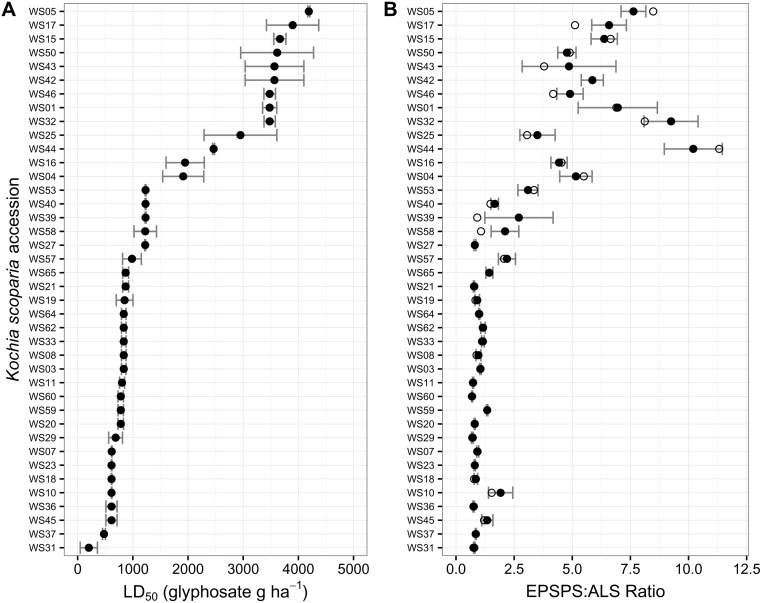
Glyphosate resistance level (A) and *EPSPS* gene copy numbers (B) for *K*. *scoparia* accessions collected from sugar beet fields by Western Sugar across a four-state region of Colorado, Nebraka, Wyoming, and Montana. Filled circles represent mean values for each accession; bars represent standard error of the mean; open circles represent median *EPSPS* gene copy number for each accession.

**Fig 2:**
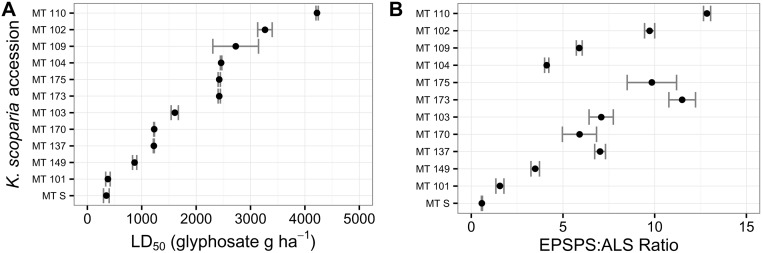
Glyphosate resistance level (A) and *EPSPS* gene copy numbers (B) for *K*. *scoparia* accessions collected from chemical fallow fields (wheat-fallow rotation) by Montana State University from Montana. Filled circles represent mean values for each accession; bars represent standard error of the mean; open circles represent median *EPSPS* gene copy number for each accession.

Estimated LD_50_ values for the WS accessions ranged from 202 to over 4000 g ae ha^-1^ with a median of 870 g ae ha^-1^ (Fig 1A). Similarly, LD_50_ values for the MT *K*. *scoparia* accessions ranged from 350 to over 4224 g ae ha^-1^ with a median LD_50_ value of 2016 (Fig 2A). Although the median LD_50_ values for both sets of *K*. *scoparia* were greater than the standard field use rate of 840 g ae ha^-1^, this is not necessarily an indication of a high level of resistance. Greater glyphosate efficacy has been observed in an outdoor environment compared to greenhouse conditions in *K*. *scoparia*, although both indoor and outdoor environments used artificial growth media rather than field soil [14]. The LD_50_ (and resulting R:S ratio) from greenhouse and field studies have been recently shown to vary in response to a variety of environmental factors when screening GR *K*. *scoparia* [22]. Because the LD_50_ can be influenced by a variety of factors, the LD_50_s in our study (or any greenhouse study) are not an absolute indicator of resistance, but rather a relative measure to compare accessions within each study.

### *EPSPS* gene copy number

**Fig 3:**
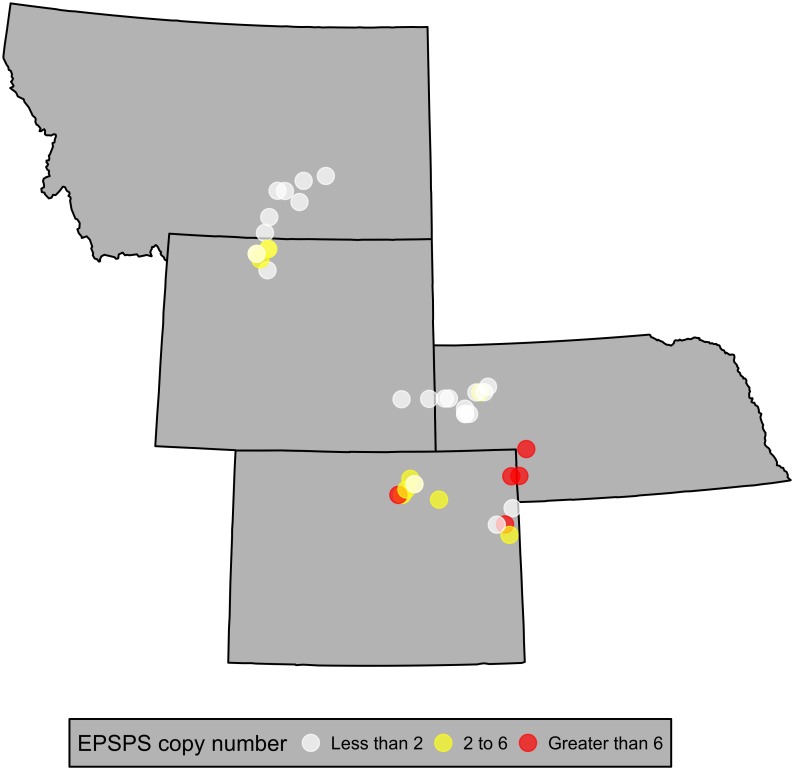
Collection locations of 40 *K*. *scoparia* accessions from the Western Sugar survey for which *EPSPS* copy numbers were quantified.

Mean *EPSPS* copy numbers in the WS *K*. *scoparia* accessions ranged from 0.7 to 10.2 (Fig 1B), and *K*. *scoparia* accessions with increased *EPSPS* copy number were identified in Wyoming, Nebraska, and Colorado (Fig 3). *EPSPS* copy numbers for the MT accessions ranged from 0.6 to 12.8 (Fig 2B). Median *EPSPS* copy numbers ranged from 0.7 to 11.3 depending on the *K*. *scoparia* accession. The median *EPSPS* copy number was less than the mean for nearly all accessions where a notable difference between median and mean was present, indicating that a few individual plants had much higher *EPSPS* copy numbers compared to the majority of plants within that accession.

### Effect of *EPSPS* copy number on whole-plant response

A notable increase in LD_50_ was apparent for accessions with median *EPSPS* value of >2.5 (Figs 1 and 2). There appeared to be a ‘plateau’ with respect to resistance level; that is, additional gene copies appeared to provide limited increase in resistance level once a certain threshold of gene copies had been reached. However, the relationship between EPSPS copy number and whole-plant response appeared more linear with the MT accessions (S1 Fig). Therefore, it is unclear whether additional *EPSPS* copies greater than 12 will continue to increase whole-plant resistance levels at a similar rate, since our samples did not include individuals with very high copy numbers.

GR *K*. *scoparia* accessions from sugarbeet fields exhibit *EPSPS* gene duplication similar to what is observed in *K*. *scoparia* from dryland chemical fallow fields (wheat-fallow rotation). Gene duplication in the tested GR samples has not risen to levels higher than 12 additional copies. Determining the *EPSPS* copy number is a valuable assay for diagnosing glyphosate resistance in *K*. *scoparia*. If a sample has increased *EPSPS* copy number (>2.5), our results suggest that the sample is GR. If a sample does not have increased *EPSPS* copy number, it is glyphosate-susceptible. However, glyphosate susceptibility can vary substantially for individual plants with a single *EPSPS* gene copy (Figs 1 and 2). In S1 File, we propose an empirical model to quantify the relationship between gene copy number and whole-plant response that accounts for this phenotypic variability.

Our overall survey results indicate that *EPSPS* copy number in GR *K*. *scoparia* from sugarbeet fields is generally within the previously observed range of 3–10. *K*. *scoparia* accessions with *EPSPS* copies <3 did not exhibit a field level of glyphosate resistance. An *EPSPS* copy number of 3 would provide approximately 2.3- to 2.9-fold level of glyphosate resistance, which may sometimes be difficult to observe under field conditions. Our analysis here is somewhat limited, since our highest median *EPSPS* copy number for any accession included in the model was 8. Additional *K*. *scoparia* accessions from eastern Colorado and Alberta, Canada with *EPSPS* copy numbers from 15 to 20 have been recently identified [23]. These populations appear to be highly resistant to glyphosate. The ability of *EPSPS* copy numbers greater than 12 to continue increasing glyphosate resistance level should be tested empirically with *K*. *scoparia* populations with higher *EPSPS* copy numbers. Inclusion of multiple low-copy individuals will aid in putting those results into context, since we have documented substantial variation in glyphosate response among low-copy number accessions.

Continued glyphosate selection pressure is likely selecting for higher *EPSPS* copy number over time, and recently, a *K*. *scoparia* population from Kansas has been confirmed to be resistant to four herbicide modes of action (PSII, ALS, glyphosate, and synthetic auxins) [6]. Known glyphosate resistance mechanisms exceed those reported for any other herbicide and include target-site mutations, target-site gene duplications, active vacuole sequestration, limited cellular uptake, and rapid necrosis response [24]. Proper stewardship of glyphosate is critical, including use of other herbicide modes of action, cultural and mechanical control practices, and preventing seed set of surviving *K*. *scoparia*.

Typically, R:S ratios at the whole-plant level are calculated using one or two ‘known susceptible’ weed biotype(s). There is little agreement in the weed science literature on what should be used as a susceptible biotype for resistance confirmation studies, and whole-plant response to glyphosate can vary widely among susceptible accessions [25]. Higher *EPSPS* gene copy number increased the estimated probability of survival in GR *Amaranthus palmeri*, when an empirical model fit to an F_2_ population segregating for *EPSPS* gene copy estimated that 53 *EPSPS* gene copies provided a 95% probability of surviving a high dose of 2000 g ae ha^-1^ glyphosate [26]. We have used a modeling approach to estimate the level of resistance expected for a single *EPSPS* copy individual, and therefore, were able to estimate resistance directly attributable to the resistance mechanism (S1 File). This method of calculating the R:S ratio should be less affected by variability in phenotypic response as a result of different genetic backgrounds. In our study, glyphosate susceptible *K*. *scoparia* (median *EPSPS* copies ≤1.5) exhibited glyphosate LD_50_ ranging from 202 to 1225 g ae ha^-1^, indicating glyphosate sensitivity can vary widely among accessions not expressing the resistance mechanism. Similar levels of variability would be expected among plants with multiple *EPSPS* copies. If a single *K*. *scoparia* accession were used as our ‘known susceptible’ biotype, the R:S ratio of our most resistant accession in the WS collection (LD_50_ = 3895) could range from 3 to 19. Using an empirical model, we provide an estimate of the level of glyphosate resistance that is attributable to the resistance mechanism and is less affected by variability contributed by other, unrelated genetic factors (S1 File). Combining predictive modeling with biological data could be a powerful approach for understanding the evolution of glyphosate resistance in *K*. *scoparia*, and we have characterized the quantitative relationship between *EPSPS* gene duplication and whole-plant glyphosate resistance level. Validation of this model will enable the diagnosis of whole-plant glyphosate resistance level in *K*. *scoparia* using the DNA marker for *EPSPS* gene copy number, a method which is much faster and less expensive than time-consuming greenhouse bioassays.

## Supporting Information

S1 Fig Glyphosate resistance level as influenced by EPSPS gene copy numbers in *K*. *scoparia* for two sets of accessions. Regression equation parameters (with standard errors in parentheses) as described in Equation 2 in S1 File: WS accessions, Rmax = 5,879 (890), K = 5.4 (1.5); MT accessions, Rmax = 21,424 (60,276), K = 66 (213). Linear regression parameters for MT accessions: slope = 255 (56); y-intercept = 243 (423).
(PNG)

S2 Fig Relative glyphosate resistance level as influenced by EPSPS gene copy numbers in *K*. *scoparia*. (A) Y-values scaled by dividing all LD_50_ values by the estimate for plants with 1 EPSPS gene copy for each set of accessions; (B) Y-values scaled by dividing all LD_50_ values by the estimate for plants with 5 EPSPS gene copies for each set of accessions.
(PNG)

S1 File Modeling the effect of EPSPS copy number on whole-plant response. (DOCX)

S1 Data Greenhouse bioassay data for WS *Kochia scoparia* accessions. Column header definitions: plot, number for experimental unit (1 pot); Rate, amount of herbicide formulated product in fluid ounces per acre; Rate.gha, rate of glyphosate in grams of acid equivalent per hectare; Accession, code given to each *K*. *scoparia* accession; Injury, visual evaluation ranging from 0 (no observable injury) to 100 (completely dead); binom95, binomial survival data (equal to 1 if Injury is less than or equal to 95 and zero if Injury is greater than 95).
(CSV)

S2 Data EPSPS gene copy data for WS *Kochia scoparia* accessions. Column header definitions: Accession, code given to each *K*. *scoparia* accession, same as S1 Data; CSU.num, identification number corresponding to laboratory studies conducted at CSU; Lat and Long, GPS coordinates truncated to 1 decimal degree; s01 through s12, EPSPS copy number for samples 1 through 12 within each accession.
(CSV)

S3 Data Greenhouse bioassay data for MT *Kochia scoparia accessions*. Column header definitions: population, code given to each *K*. *scoparia* accession; fl.ozA, glyphosate rate applied expressed in fluid ounces per acre of formulated product; rate.gha, glyphosate rate applied expressed in grams of acid equivalent per hectare; vis.control, visual evaluation ranging from 0 (no observable injury) to 100 (completely dead); mort.95, binomial survival data (equal to 1 if Injury is less than or equal to 95 and zero if Injury is greater than 95).
(CSV)

S4 Data EPSPS gene copy data for MT *Kochia scoparia* accessions. Column header definitions: population, code given to each *K*. *scoparia* accession (same as S3 Data); EPSPScopy, EPSPS gene copy number for each sample; EPSPS.se, standard error associated with the gene copy number for each sample.
(CSV)
