# Supplementary figures and images for: EPSPS Gene Copy Number and Whole-Plant Glyphosate Resistance Level in Kochia scoparia
Source: PLoS One. 2016 Dec 16;11(12):e0168295. doi: 10.1371/journal.pone.0168295 (PMC5161467; doi:10.1371/journal.pone.0168295)

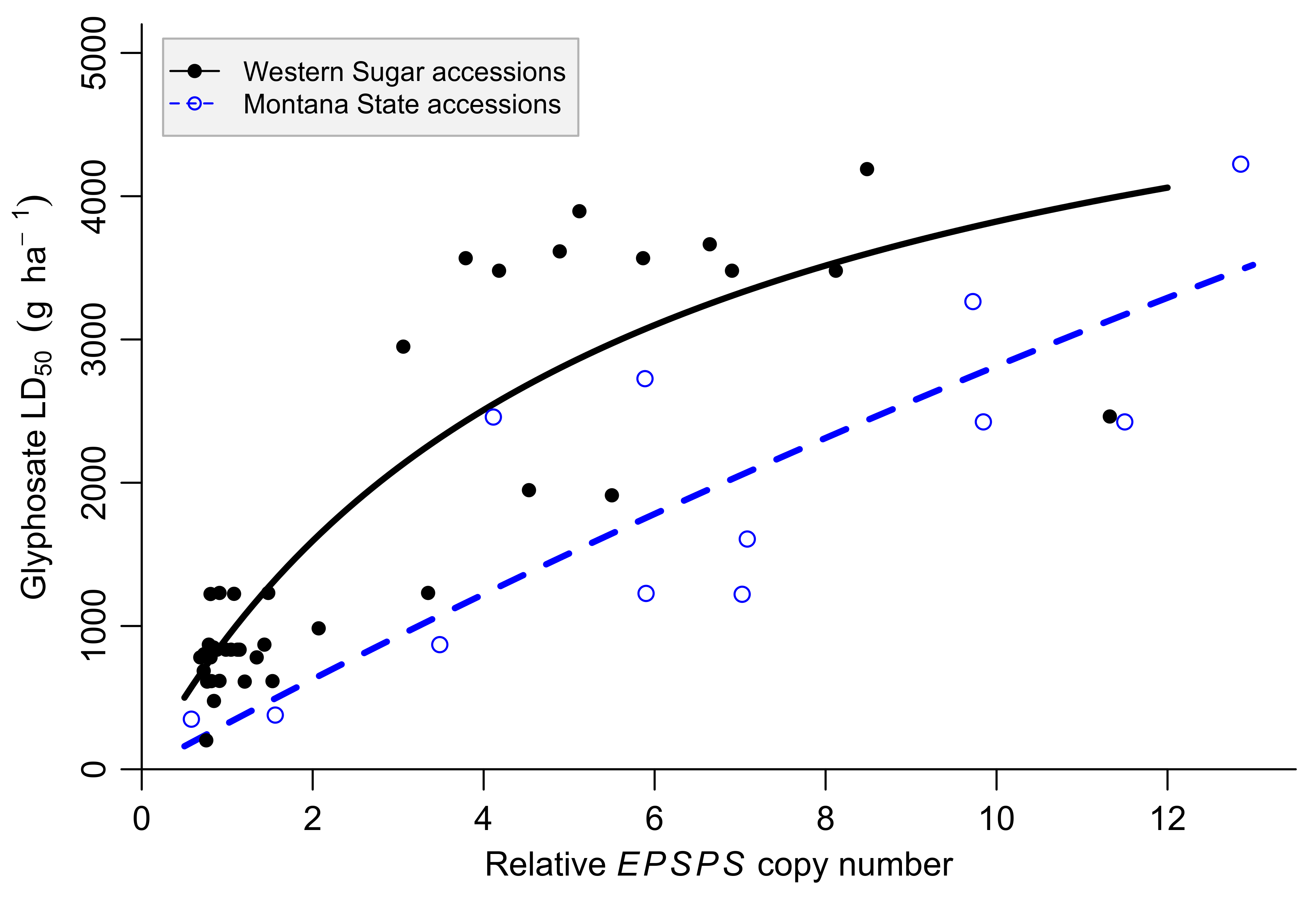

Supplement: S1 Fig — Regression equation parameters (with standard errors in parentheses) as described in Equation 2 in S1 File: WS accessions, Rmax = 5,879 (890), K = 5.4 (1.5); MT accessions, Rmax = 21,424 (60,276), K = 66 (213). Linear regression parameters for MT accessions: slope = 255 (56); y-intercept = 243 (423). (PNG) [file pone.0168295.s001.png]

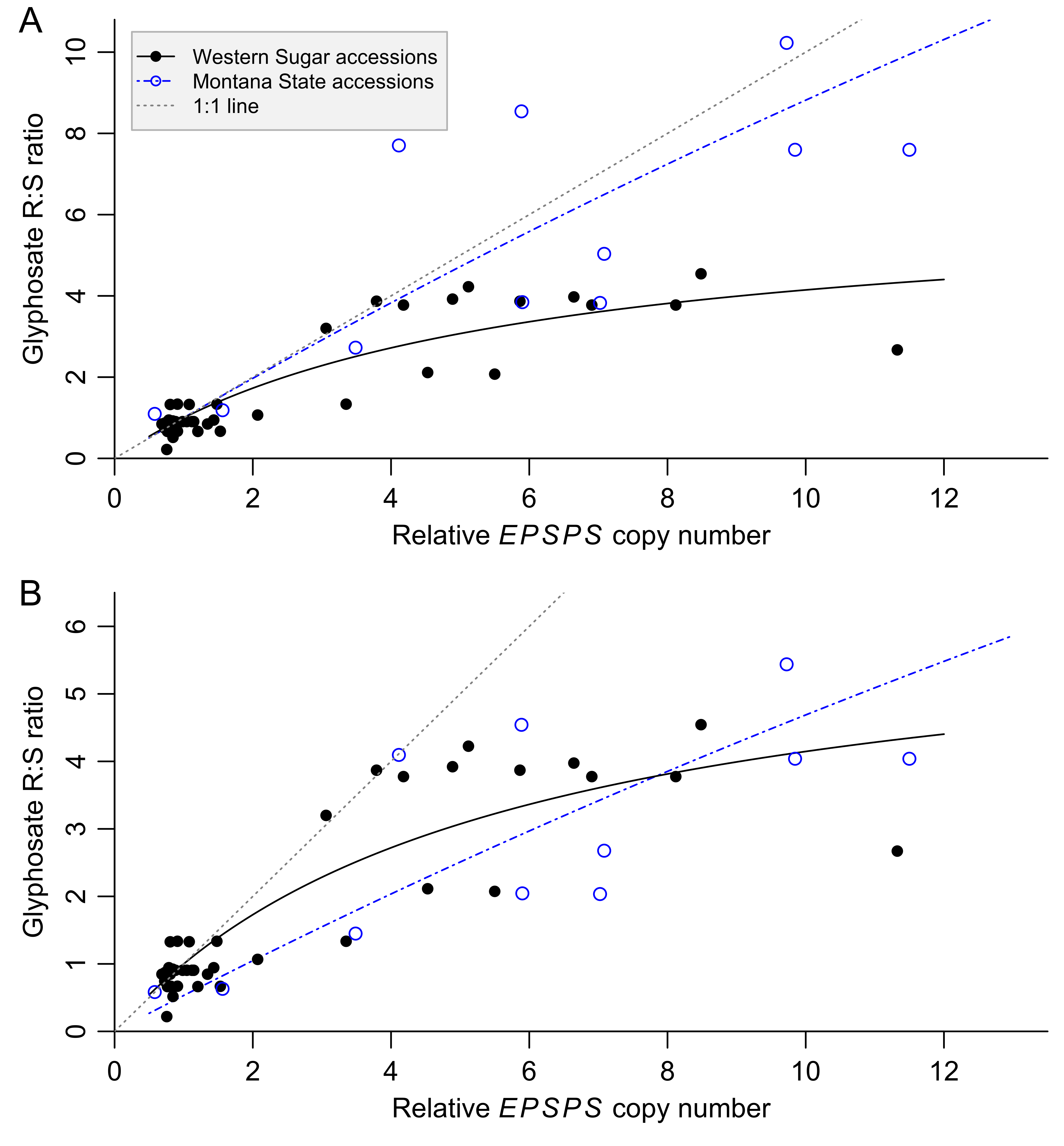

Supplement: S2 Fig — (A) Y-values scaled by dividing all LD50 values by the estimate for plants with 1 EPSPS gene copy for each set of accessions; (B) Y-values scaled by dividing all LD50 values by the estimate for plants with 5 EPSPS gene copies for each set of accessions. (PNG) [file pone.0168295.s002.png]
