# Supplementary material for: EPSPS Gene Copy Number and Whole-Plant Glyphosate Resistance Level in Kochia scoparia
Source: PLoS One. 2016 Dec 16;11(12):e0168295. doi: 10.1371/journal.pone.0168295 (PMC5161467; doi:10.1371/journal.pone.0168295)
Supplement: S1 File — (DOCX) [file pone.0168295.s003.docx]

**Modeling the effect of EPSPS copy number on whole-plant response**

**Methods:**

The relationship between LD_50_ from the greenhouse bioassay and the median *EPSPS* copy number for each accession was quantified using regression. Because the dose-response study was conducted at the accession level (that is, multiple individuals from each accession were used as experimental units), the median *EPSPS* value was used as the independent variable to quantify the relationship between gene copy number and whole-plant resistance. The mean copy number could be skewed significantly by a few (or even one) high copy number individuals within a population, and therefore, overestimate the copy number for an accession.

We did not know *a priori* which type of regression model was appropriate to quantify the relationship between *EPSPS* gene copy number and whole-plant glyphosate resistance. A linear regression would be theoretically appropriate only if there is no limitation to the amount of glyphosate *K. scoparia* could survive, as long as *EPSPS* gene copy number could be sufficiently increased. However, if there is a limitation to the effectiveness of additional *EPSPS* copies in increasing glyphosate resistance levels, then an asymptotic model should be more appropriate. Based on a preliminary visual evaluation, three different models were fit to the data; a three-parameter rectangular hyperbolic model, a two-parameter rectangular hyperbolic model, and a simple linear model. The three-parameter model was of the form:

Equation 2: *Y*=*L*+((*R_max_*-*L*)/(1+*K*/*X*))

In Equation 2, *Y* is the LD_50_ from the greenhouse bioassay; *X* is the *EPSPS* copy number; *R_max_* is an upper asymptote, or the maximum theoretical LD_50_ at very high values of *X*; *L* is the estimated LD_50_ when *X*=0; and *K* is the value of *X* that results in *Y* halfway between *L* and *R_max_*. The two-parameter nonlinear model was the same, but with *L=0*; setting *L*=0 reduced the equation to the Michaelis-Menten model. The linear model used was a simple linear regression. After all three models were fit to the data, Akaike information criterion (AIC) and visual assessment was used to determine which model provided the best fit to the data for the WS and MT *K. scoparia* accessions.

**Results:**

Based on AIC, the 2 parameter Michaelis-Menten model fit was chosen as the best model (of those tested) to describe the relationship between resistance level and *EPSPS* gene copy number for the WS *K. scoparia* accessions. However, AIC values for the linear model and two-parameter nonlinear model were nearly identical for the MT *K. scoparia* accessions.

*R_max_*, or the theoretical maximum LD_50_ when the number of *EPSPS* gene copies is very large, was 5879 g ae ha^-1^ for the WS accessions, and 21,424 g ae ha^-1^ for the MT accessions (S1 Fig). The *R_max_* parameter from greenhouse studies, as an estimate of LD_50_, is not very useful in absolute terms. The LD_50_ value depends heavily on the environmental conditions during the bioassay [1, 2], and can vary significantly from one experimental run to the next even when using the same genotypes and experimental design. However, the LD_50_ ratio between resistant and susceptible biotypes tends to remain relatively more stable than other responses such as GR_50_ calculated from dry weight [3]. A standardized estimate of resistance level can therefore be calculated by dividing the *R_max_* parameter by the model estimate of LD_50_ for a plant with a single *EPSPS* gene. Practically speaking, this gives an estimate of the maximum “fold” resistance that would be expected due to increased *EPSPS* gene copies. For the WS accessions, the estimated LD_50_ for accessions with 1 *EPSPS* copy was 922 g ae ha^-1^, suggesting the maximum resistance level would be approximately 6.4-fold (5879 / 922). This estimated maximum level of glyphosate resistance is similar to previous work on glyphosate resistance, which report between 4- to 11-fold resistance in glyphosate-resistant *K. scoparia* accessions compared to a single susceptible accession [2, 4, 5]. For the MT accessions, however, the *R_max_* for the nonlinear model is 21,424 g ae ha^-1^, and the LD_50_ estimate for a single copy is 319 g ae ha^-1^, making the estimated maximum resistance level 64-fold based on these accessions.


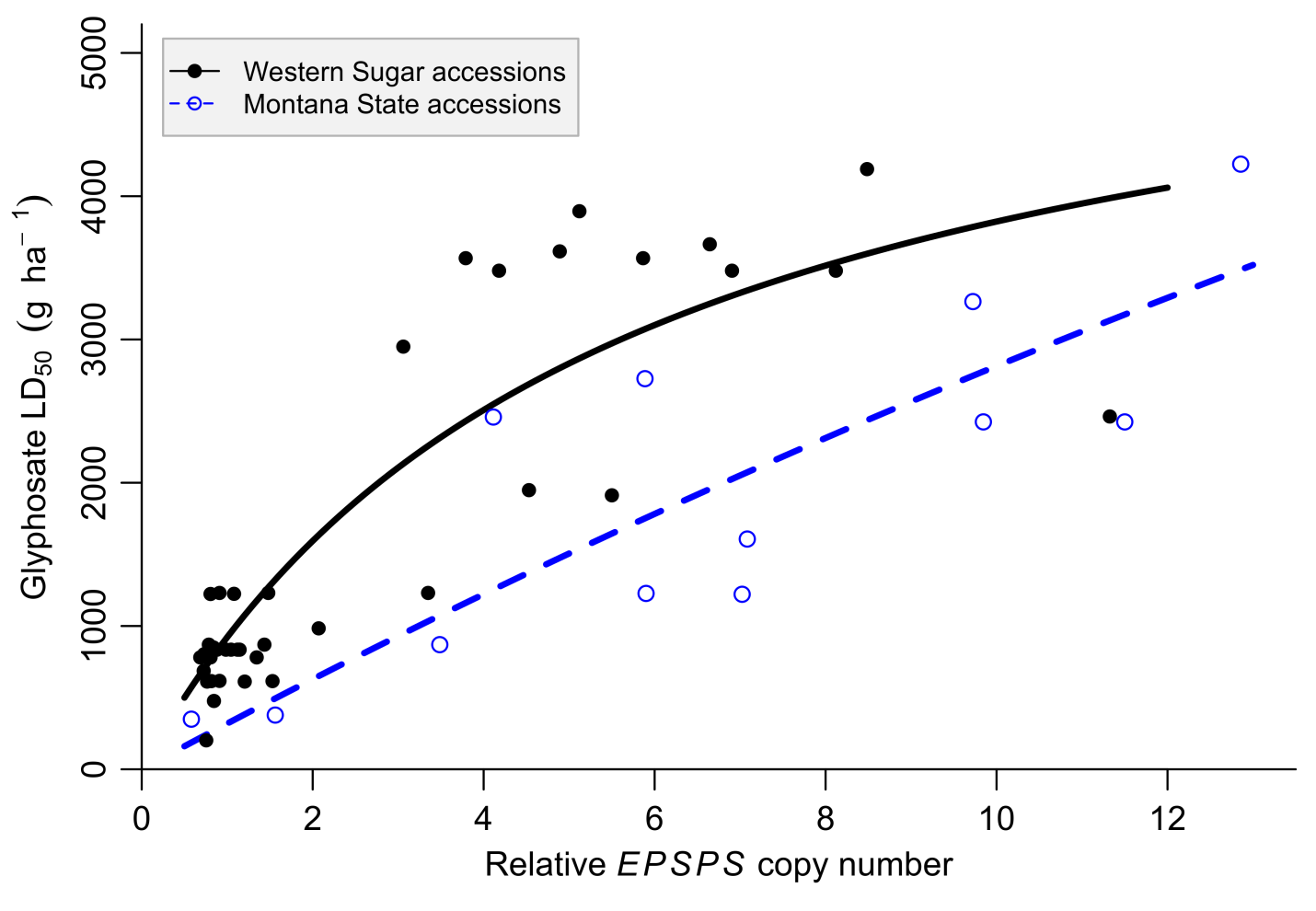


**S1 Fig. Glyphosate resistance level as influenced by *EPSPS* gene copy numbers in *K. scoparia* for two sets of accessions.** Regression equation parameters (with standard errors in parentheses) as described in Equation 3: WS accessions, *R_max_* = 5,879 (890), *K* = 5.4 (1.5); MT accessions, *R_max_* = 21,424 (60,276), *K* = 66 (213). Linear regression parameters for MT accessions: slope = 255 (56); y-intercept = 243 (423).

The difference between the two sets of *K. scoparia* accessions may be less due to the maximum resistance level than the estimate of a “wild-type” resistance level (*EPSPS* copy number of 1). This can be illustrated by re-scaling the data so that the y-axis is the R:S ratio rather than raw LD_50_ by dividing each LD_50_ by the estimated value for 1 *EPSPS* copy within each group of accessions (S2 Fig). In this relative format, the MT accessions appear far more resistant than the WS accessions for any given number of *EPSPS* copy numbers. The MT data set only includes two *K. scoparia* accessions with low-copy numbers, compared to many accessions in the WS data set. Therefore, we are more confident in the low copy-number LD_50_ estimates in the WS data set. If the y-axis is instead scaled so that the MT data is similar to the WS accessions for 5 *EPSPS* gene copies (the 25^th^ percentile, where we have more confidence in the relationship), the relative differences between the two sets of accessions is minimal (S2 Fig).

Our approach is different from many previous calculations of R:S ratio, since we included many *K. scoparia* accessions that exhibited a susceptible response. This allows a more robust estimation of the variability in whole-plant response among susceptible accessions, which then allows a more accurate estimate of the R:S ratio at the whole plant level.


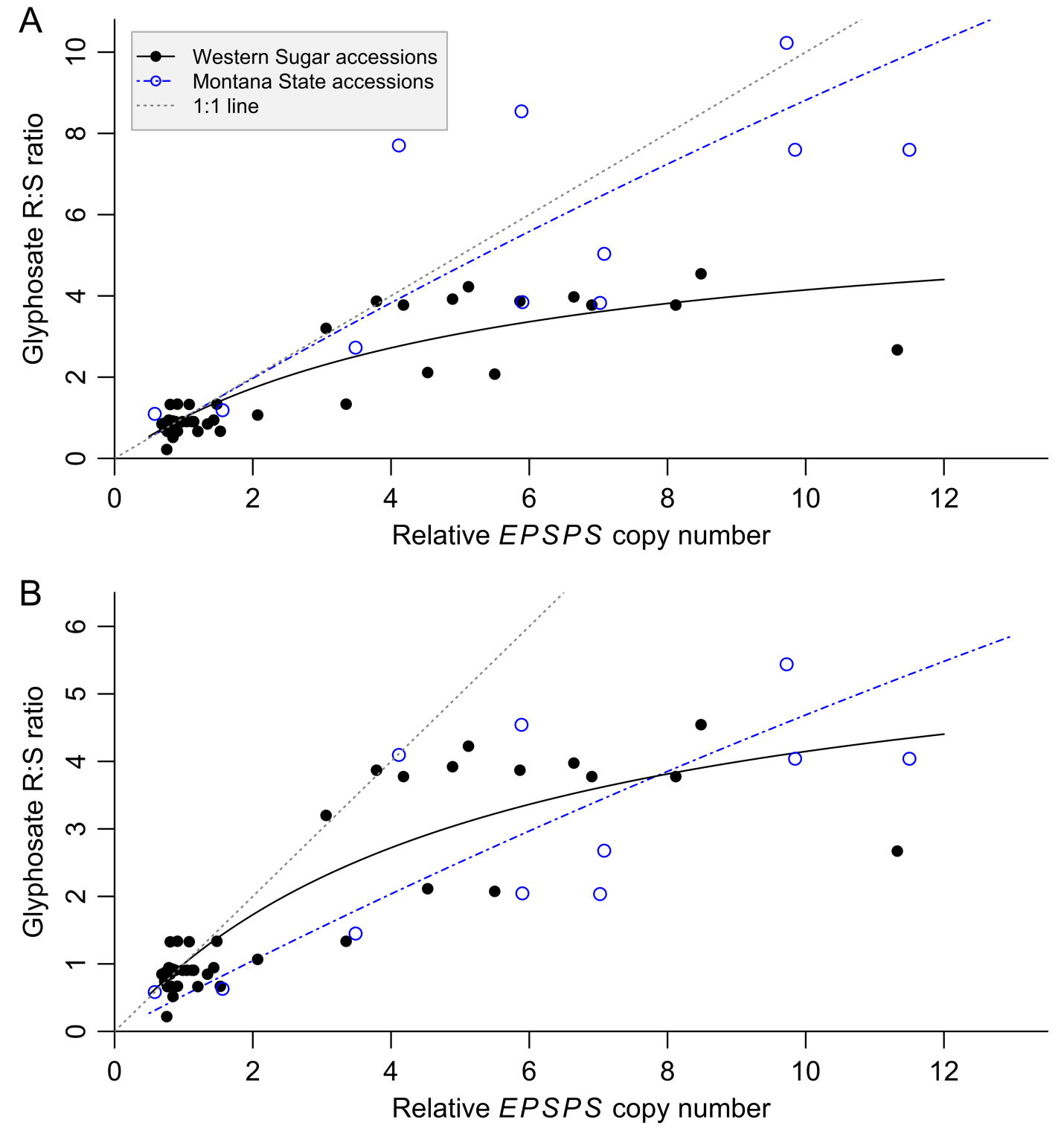


**S2 Fig. Relative glyphosate resistance level as influenced by *EPSPS* gene copy numbers in *K. scoparia***. (A) Y-values scaled by dividing all LD50 values by the estimate for plants with 1 *EPSPS* gene copy for each set of accessions; (B) Y-values scaled by dividing all LD50 values by the estimate for plants with 5 *EPSPS* gene copies for each set of accessions.

**References**

1. Kudsk P, Jensen PK. Prediction of herbicide activity. Weed Res. 1988;28(6):473-8.

2. Godar AS, Stahlman PW, Jugulam M, Dille JA. Glyphosate-resistant kochia (*Kochia scoparia*) in Kansas: EPSPS gene copy number in relation to resistance levels. Weed Sci. 2015;63(3):587-95.

3. Coburn C, Kniss AR. Influence of experimental methods on R:S ratio in herbicide resistance studies. Proc Western Soc Weed Sci. 2015;68:124.

4. Beckie HJ, Blackshaw RE, Low R, Hall LM, Sauder CA, Martin S, et al. Glyphosate- and acetolactate synthase inhibitor–resistant kochia (*Kochia scoparia*) in Western Canada. Weed Sci. 2013;61(2):310-8.

5. Kumar V, Jha P, Reichard N. Occurrence and characterization of kochia (*Kochia scoparia*) accessions with resistance to glyphosate in Montana. Weed Technol. 2014;28(1):122-30.
